# Lung hyperinflation by mechanical ventilation versus isolated tracheal aspiration in the bronchial hygiene of patients undergoing mechanical ventilation

**DOI:** 10.5935/0103-507X.20160010

**Published:** 2016

**Authors:** Crisiela Brum Assmann, Paulo José Cardoso Vieira, Fernanda Kutchak, Marcelo de Mello Rieder, Soraia Genebra Ibrahim Forgiarini, Luiz Alberto Forgiarini Junior

**Affiliations:** 1 Centro Universitário Metodista - IPA - Porto Alegre (RS), Brazil.; 2 Hospital Cristo Redentor - Porto Alegre (RS), Brazil.; 3 Universidade do Vale dos Sinos - Porto Alegre (RS), Brazil.

**Keywords:** Respiration, artificial, Suction, Pulmonary ventilation, Intensive care units

## Abstract

**Objective:**

To determine the efficacy of lung hyperinflation maneuvers via a mechanical ventilator compared to isolated tracheal aspiration for removing secretions, normalizing hemodynamics and improving lung mechanics in patients on mechanical ventilation.

**Methods:**

This was a randomized crossover clinical trial including patients admitted to the intensive care unit and on mechanical ventilation for more than 48 hours. Patients were randomized to receive either isolated tracheal aspiration (Control Group) or lung hyperinflation by mechanical ventilator (MVH Group). Hemodynamic and mechanical respiratory parameters were measured along with the amount of aspirated secretions.

**Results:**

A total of 50 patients were included. The mean age of the patients was 44.7 ± 21.6 years, and 31 were male. Compared to the Control Group, the MVH Group showed greater aspirated secretion amount (3.9g versus 6.4g, p = 0.0001), variation in mean dynamic compliance (-1.3 ± 2.3 versus -2.9 ± 2.3; p = 0.008), and expired tidal volume (-0.7 ± 0.0 versus -54.1 ± 38.8, p = 0.0001) as well as a significant decrease in peak inspiratory pressure (0.2 ± 0.1 versus 2.5 ± 0.1; p = 0.001).

**Conclusion:**

In the studied sample, the MVH technique led to a greater amount of aspirated secretions, significant increases in dynamic compliance and expired tidal volume and a significant reduction in peak inspiratory pressure.

## Abstract (pt)

**Objetivo:**

Determinar a eficácia da manobra de hiperinsuflação pulmonar com o ventilador mecânico, em comparação à aspiração traqueal isolada, para remover secreções, normalizar a hemodinâmica e melhorar a mecânica pulmonar em pacientes em ventilação mecânica.

**Métodos:**

Ensaio clínico randomizado cruzado incluindo pacientes em ventilação mecânica por mais de 48 horas internados na unidade de terapia intensiva. Os pacientes foram randomizados para receber a aspiração traqueal isolada (Grupo Controle) e hiperinsuflação pulmonar por meio do ventilador mecânico (Grupo HVM). Mensuraram-se parâmetros hemodinâmicos e de mecânica respiratória, assim como a quantidade de secreção aspirada.

**Resultados:**

Foram incluídos 50 pacientes. A média de idade dos pacientes foi de 44,7 ± 21,6 anos, sendo 31 do sexo masculino. O Grupo HVM apresentou maior quantidade de secreção aspirada (3,9g *versus* 6,4g; p = 0,0001), variação na média da complacência dinâmica (-1,3 ± 2,3 *versus* -2,9 ± 2,3; p = 0,008), volume corrente expirado (-0,7 ± 0,0 *versus* -54,1 ± 38,8; p = 0,0001) e diminuição significativa da pressão inspiratória de pico (0,2 ± 0,1 *versus* 2,5 ± 0,1; p = 0,001), em comparação com o Grupo Controle.

**Conclusão:**

Na amostra estudada, a técnica de HVM apresentou maior quantidade de secreção aspirada, aumento significativo da complacência dinâmica e volume corrente expirado, além de diminuição significativa da pressão de pico inspiratória.

## INTRODUCTION

Mechanical ventilation (MV) aims to reverse or prevent respiratory muscle fatigue, reduce muscular work and consumption of oxygen and maintain gas exchange. MV also thereby reduces respiratory distress and allows specific treatments to be applied.^(1)^

Patients are subject not only to the benefits of this support but also to various risk factors, such as the development of mechanical ventilation-associated pneumonia (VAP).^(2)^ VAP is one of the main factors contributing to increases in mortality, length of stay in the intensive care unit (ICU), overall hospitalization time and health-related costs.^(2,3)^

Tracheal intubation, immobility imposed on the patient for sedation and general weakness with diminished cough effectiveness reduce mucociliary transport and promote the retention of secretions in the airway.^(2,4)^ Lung secretion buildup can cause increased airway resistance and partial or total airway obstruction, with consequent alveolar hypoventilation and the development of atelectasis and hypoxemia and increased effort needed to breathe.^(5,6)^ The care of these patients includes tracheal aspiration, which is used to facilitate the removal of secretions from the airway. However, when applied alone, tracheal aspiration can be ineffective and may clear only a small portion of the airway.^(7)^

There are some physical therapy techniques aimed at bronchial hygiene and that thus prevent bronchial obstruction by secretion buildup. Among these techniques are the use of positive pressure devices, including lung hyperinflation using a mechanical ventilator (MVH).^(8)^ This technique consists of the administration of high tidal volumes, either by progressively increasing support pressure until a peak pressure of 40cmH_2_O is achieved in the airway or by increasing positive end-expiratory pressure (PEEP).^(9)^ MVH promotes the expansion of collapsed alveoli, increasing air flow to areas with atelectasis through collateral channels and surfactant renewal in the alveoli. This technique also aims to increase the elastic potential of lung recoil and peak expiratory flow, resulting in the mobilization of lung secretions from the periphery of the lungs to more central regions.^(9-11)^

The objective of this study was to determine the efficacy of lung hyperinflation maneuvers using a mechanical ventilator compared to isolated tracheal aspiration for removing secretions, normalizing hemodynamics and improving lung mechanics in patients on mechanical ventilation.

## METHODS

This was a randomized crossover clinical trial developed in the ICU of the *Hospital Cristo Redentor*, which belongs to the *Grupo Hospitalar Conceição* in Porto Alegre (RS), Brazil. The study was approved by the *Centro Universitário Metodista* (IPA) Research Ethics Committee under protocol number 1,048,322. All the responsible parties for the participating patients signed a free and informed consent form.

**Figure 1:**
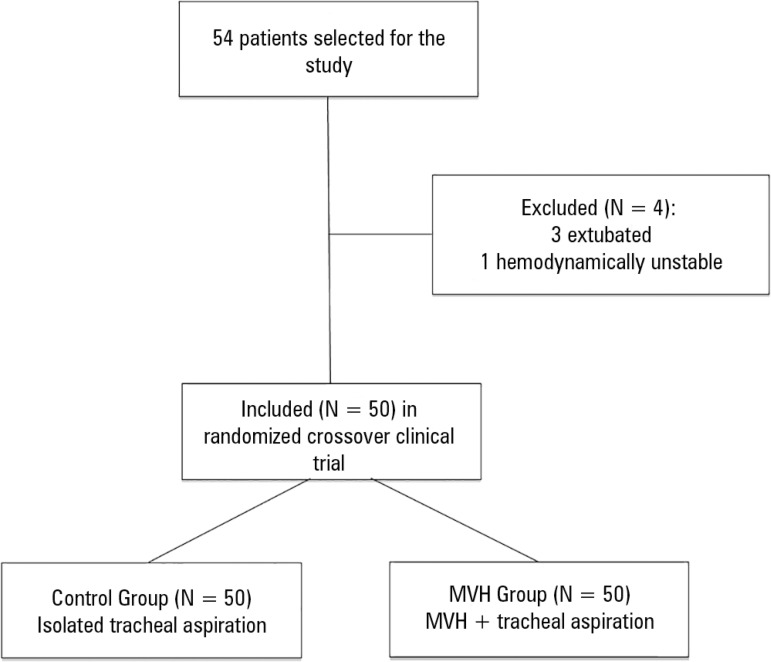
Flowchart of patients included in the study. MVH - lung hyperinflation by mechanical ventilation.

From May to September 2015, all ICU patients who were on MV for more than 48 hours without a diagnosis of VAP and with PEEP ≤ 10cmH_2_O and who had undergone aspiration 2 hours before application of the protocol and were hemodynamically stable (mean arterial pressure ≥ 60 and ≤ 120mmHg) were included in the study. Patients with contraindications for increased positive pressure were excluded, such as those with undrained pneumothorax and hemothorax or subcutaneous emphysema, those with peak pressure > 40cmH_2_O and neurosurgical patients. Thus, 54 patients were initially included in the study; 4 were then excluded, 3 due to being extubated before the conclusion of the protocol and 1 due to hemodynamic instability (Figure 1).

All 50 patients who met the inclusion criteria were evaluated and randomized into groups receiving isolated tracheal aspiration (Control Group) or lung hyperinflation by mechanical ventilation (MVH Group). Randomization was performed using blocks of sealed envelopes allocating each patient to the first technique to be performed. After 24 hours, the patient underwent the other technique; thus, all patients underwent both techniques.

Aspiration was performed 2 hours before the patient underwent each technique to equate the groups in relation to secretion volumes. To this end, patients were placed in the supine position with the head elevated to 30º and were subjected to aspiration on a single occasion (probe number 12; MarkMed^®^ Ind e Com. Ltda, São Paulo, Brazil) with a vacuum adjusted to a pressure of -40cmH_2_O.

The patients randomized to the Control Group were ventilated for 1 minute with a fraction of inspired oxygen (FiO_2_) of 100%. Each patient was then removed from the ventilator and subjected to three rounds of aspiration, each lasting 15 seconds. The aspirated secretions were stored in a collection bottle (Intermedical^®^; Intermedical - Setmed, São Paulo, Brazil). Changes in hemodynamic and lung parameters were recorded prior to the application of the technique and immediately after the aspirations.

Patients randomized to the MVH Group were placed in the supine position with the head elevated to 30º. In ventilatory pressure mode, inspiratory pressure was increased by 10cmH_2_O. In volume ventilation mode, the ideal tidal volume of each patient was calculated, and the tidal volume was consequently increased by 50% for a 10-minute period while ensuring that the peak inspiratory pressure (PIP) did not exceed 40cmH_2_O. The patients were subjected to aspiration, and secretions were collected in the same manner as for the control group patients.

Before and immediately after the application of each technique, lung and hemodynamic parameters were evaluated. In both groups, the aspirate was weighed in the same manner by a blinded operator using a high-precision balance (E.clear, Origin: PCR, Import: Bravo Brasil Com. Imp. Exp. Ltd.).

Hemodynamic parameters such as heart rate, respiratory rate, mean arterial pressure and peripheral oxygen saturation were recorded using a multiparameter monitor (Infinity^®^ Kappa, Dräger, Germany). Respiratory function was evaluated before and after the techniques were applied by measuring PIP, expired tidal volume (ETV) and dynamic compliance (Cdyn). The delta values ​​(Δ) were calculated as the difference between the initial and final lung hemodynamic parameters.

### Statistical analysis

All continuous data are presented as means and standard deviations, and categorical data are presented as absolute values and percent frequencies. Normality was evaluated using the Shapiro-Wilk test*.* Student's *t*-test for paired measures was used to compare groups, and intergroup comparisons were performed using Student's *t*-test for independent measures. All data were stored and analyzed using the Statistical Package for the Social Sciences (SPSS) for Windows, version 17.0, and a significance level of 0.05 was adopted.

## RESULTS

The study included 50 individuals who were treated between May and September 2015. There was a predominance of male patients. The patients' mean age was 44.7 ± 21.6 years, and the predominant pathology was traumatic brain injury (Table 1).

**Table 1:** Clinical characteristics of the sample

**Variable**	**N = 50**
Age (years)	44.7 ± 21.6
Male	31 (62)
Conditions	
Brain injury	8 (16)
Burn	6 (12)
Stab wound	5 (10)
Subarachnoid hemorrhage	7 (14)
Fracture	4 (8)
Stroke	4 (8)
Brain tumor	3 (6)
Intracerebral hemorrhage	3 (6)
Others*	10 (20)

* Others - septic shock, focal trauma, acute myocardial infarction, intra-abdominal organ trauma, hydrocephalus and sensory loss, head trauma, firearm injury and diffuse paresis. The results are expressed as the mean ± standard deviation or as number (%).

Significant differences in hemodynamic variables were observed between the Control Group and the MVH Group. There were significant increases in Cdyn and ETV in the MVH Group compared to the Control Group. In the MVH Group, ETV also increased significantly immediately after the intervention compared to the period before the intervention.

There was a significant reduction in PIP variation in the MVH Group compared to the controls. No other variations in the analyzed parameters differed significantly between the groups (Table 2).

**Table 2:** Comparison of variations in hemodynamic and lung parameters in the sample

	**Control Group**			**MVH Group**			**p-value**
	**Pre**	**Post**	**Δ (Pre - Post)**	**Pre**	**Post**	**Δ (Pre - Post)**	
Cdyn (cmH_2_O)	31.4 ± 6.5	32.7 ± 8.8	-1.3 ± 2.3	30.0 ± 11.5	32.9 ± 9.2	-2.9 ± 2.3	0.008
PIP (cmH_2_O)	22.7 ± 3.7	20.9 ± 3.7	0.2 ± 0.1	24.3 ± 5.4	21.8 ± 5.4	2.5 ± 0.1	0.001
AR	18.2 ± 4.7	15.6 ± 2.2	2.6 ± 2.5	18.6 ± 5.2	15.6 ± 2.7	3.0 ± 2.5	0.425
ETV (mL)	498.3 ± 62.7	499.0 ± 62.7	-0.7 ± 0.0	447.9 ± 105.2	502.0 ± 144.0*	-54.1 ± 38.8	0.0001
RF (irpm)	20.7 ± 6.9	20.6 ± 6.3	0.1 ± 0.6	17.8 ± 3.1	22.0 ± 10.9	4.2 ± 7.8	0.310
MAP (mmHg)	102.7 ± 18.6	96.8 ± 18.5	5.9 ± 0.1	95.6 ± 25.1	95.0 ± 20.1	0.6 ± 5.0	0.521
HR (bpm)	92.0 ± 12.7	99.8 ± 21.2	-7.8 ± 8.5	89.6 ± 15.5	89.4 ± 17.6	0.2 ± 2.1	0.453
SpO_2_ (%)	97.6 ± 1.3	97.6 ± 2.5	0.0 ± 1.2	98.4 ± 1.3	98.7 ± 1.5	-0.3 ± 0.2	0.769

MVH - lung hyperinflation by mechanical ventilation; Cdyn - dynamic compliance; PIP - peak inspiratory pressure; AR - airway resistance; ETV - expired tidal volume; RF - respiratory frequency; MAP - mean arterial pressure; HR - heart rate; SpO2 - peripheral oxygen saturation.

* p = 0.03.

**Figure 2:**
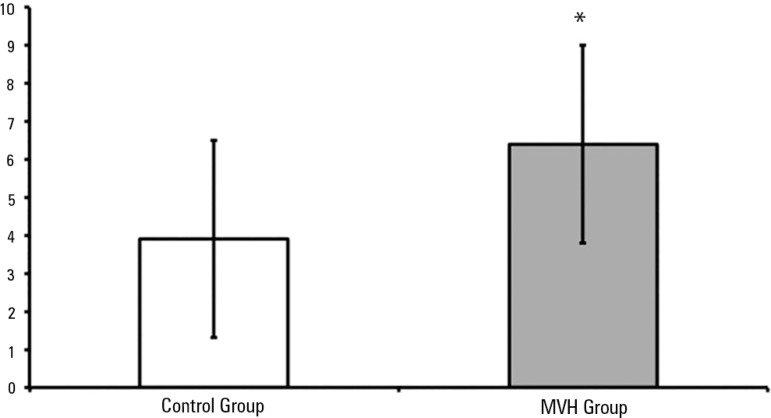
Amounts of aspirated secretions in the control and MVH Groups, * p < 0.0001 (aspiration 3.9 ± 2.6 and MVH 6.4 ± 2.6). MVH - lung hyperinflation by mechanical ventilation.

A significantly greater mean amount of secretions was aspirated in the MVH Group compared to the Control Group (p = 0.0001; Figure 2).

## DISCUSSION

In this study, the use of MVH by applying increased support pressure of 10cmH_2_O or 50% tidal volume over a period of 10 minutes resulted in increases in Cdyn and ETV and a reduction in PIP. This technique also led to greater aspiration of secretions compared with the Control Group.

The male gender was predominant (62% of the sample). This finding corroborates published reports addressing the profiles of patients admitted to Brazilian ICU.^(12)^ Among the conditions encountered, head injuries were found in 38% of the sample, which is explained by the study site being a trauma reference hospital. This finding is similar to that of an ICU with the same clinical profile.^(13)^

Studies show that the lung hyperinflation technique in mechanically ventilated critically ill patients provides increased secretion removal, re-expansion of atelectatic areas and improved lung compliance and oxygenation.^(14,15)^ This technique can be performed either by the manual hyperinflation technique (delivering a higher base tidal volume and a peak airway pressure of up to 40cmH_2_O using a manual resuscitator) or by MVH (changing the ventilation parameters on the mechanical ventilator). The ideal tidal volume for each patient should be calculated assuming 6mL/kg of predicted weight, and to ensure a protective ventilatory strategy, this value should be re-evaluated according to the individual's clinical evolution. Physical therapy techniques that involve high tidal volumes should apply a maximum airway pressure of 40cmH_2_O to avoid barotrauma.^(16,17)^ It is important to note that the present study is one of the few in the literature to compare the MVH technique in isolation with tracheal aspiration; reports of associated techniques in such a procedure are common.^(8,9)^

In this study, the MVH technique increased ETV, Cdyn, and the volume of aspirated secretions compared to the Control Group. In a randomized crossover clinical trial with 34 patients on MV that compared isolated aspiration to MVH combined with the chest compression maneuver, the latter produced a greater amount of aspirated secretions, tidal volume (VT) and Cdyn compared with isolated aspiration.^(9)^ Lemes et al. compared MVH in the lateral decubitus position to tracheal aspiration in the same position in 30 mechanically ventilated patients and found a greater amount of aspirated secretions and increased lung compliance in the former group. The increase in lung compliance is related to the re-expansion of collapsed alveoli, resulting in lung hyperinflation, which better distributes airflow.^(8)^

The increase in ETV that was demonstrated after application of the MVH technique may be related to the increase in airway pressure, which consequently generates increased lung volume. The increased ETV may also be the result of secretion removal, which reduces airway resistance and hence increases lung volume.^(5,8)^

The significant increase in Cdyn in the MVH Group observed in this study is supported by the literature^(8,9,16)^ and may result from the opening of collapsed lung units.

The use of this technique was evaluated by Dennis et al., who conducted a prospective study evaluating the prevalence of MVH use by physical therapists in Australian ICU. Only 35% of the ICU used MVH, and the leading cause of non-use of the technique was a lack of training and knowledge. An important point is that MVH was applied by professionals in both spontaneous and controlled ventilation modes, similar to this study.^(18)^

An alternative to MVH is manual hyperinflation, which produces similar results. Dennis et al. compared both techniques in a randomized crossover clinical trial with 46 patients. Their findings revealed no significant difference between the techniques in terms of volume of aspirated secretions, indicating that MVH has the same efficacy and safety as manual hyperinflation but has the advantage that the ventilator is not disconnected during execution of the technique.^(15)^ Another advantage of MVH over manual hyperinflation is the possibility of maintaining PEEP levels; the literature shows that for displacement of secretions from the distal to central airways, the peak expiratory flow should be 10% higher than the peak inspiratory flow. MVH can also prevent contamination associated with disconnecting the ventilator circuit from the patient. Thus, in cases of increased PEEP and FiO_2_, MVH should be preferentially applied over the manual hyperinflation technique.^(14,18)^

## CONCLUSION

Lung hyperinflation using a ventilator as opposed to isolated tracheal aspiration in mechanically ventilated patients resulted in an increased amount of aspirated secretions. Following application of this technique, significant increases in expired tidal volume and dynamic compliance were recorded along with a significant decrease in peak inspiratory pressure.
